# The change of non-alcoholic fatty liver disease is associated with risk of incident diabetes

**DOI:** 10.3389/fendo.2023.1108442

**Published:** 2023-05-04

**Authors:** Congling Chen, Yuecheng Zhang, Yujuan Fan, Zhen Ying, Qing Su, Xiaoying Li, Li Qin

**Affiliations:** ^1^ Department of Endocrinology and Metabolism, Zhongshan Hospital, Fudan University, Shanghai, China; ^2^ General Practice Department, Affiliated Kunshan Hospital of Jiangsu University, Suzhou, China; ^3^ Department of Endocrinology and Metabolism, Xinhua Hospital, Shanghai Jiaotong University School of Medicine, Shanghai, China; ^4^ Department of Endocrinology, Chongming Hospital Affiliated to Shanghai University of Health & Medicine Sciences, Shanghai, China

**Keywords:** non-alcoholic fatty liver disease, incident diabetes, obesity, type2 diabetes mellitus (T2DM), prevention

## Abstract

**Background & aims:**

The effect of change in non-alcoholic fatty liver disease (NAFLD) status on incident diabetes has not been well studied. We aimed to investigate the association of NAFLD development and remission with the risk of incident diabetes during a median of 3.5-year follow-up.

**Methods:**

A total of 2690 participants without diabetes were recruited in 2011-2012 and assessed for incident diabetes in 2014. Abdominal ultrasonography was used to determine the change of NAFLD. 75 g oral glucose tolerance test (OGTT) was performed to determine diabetes. NAFLD severity was assessed using Gholam’s model. The odds ratios (ORs) for incident diabetes were estimated by logistic regression models.

**Results:**

NAFLD was developed in 580 (33.2%) participants and NAFLD remission occurred in 150 (15.9%) participants during a median of 3.5-year follow-up. A total of 484 participants developed diabetes during follow-up, including 170 (14.6%) in consistent non-NAFLD group, 111 (19.1%) in NAFLD developed group, 19 (12.7%) in NAFLD remission group, and 184 (23.2%) in sustained NAFLD group. The development of NAFLD increased the risk of incident diabetes by 43% (OR, 1.43; 95%CI, 1.10-1.86) after adjustment for multiple confounders. Compared with sustained NAFLD group, remission of NAFLD reduced the risk of incident diabetes by 52% (OR, 0.48; 95%CI, 0.29-0.80). The effect of NAFLD alteration on incident diabetes was not changed after adjustment for body mass index or waist circumference, change of body mass index or waist circumference. In NAFLD remission group, participants with non-alcoholic steatohepatitis (NASH) at baseline were more likely to develop diabetes (OR, 3.03; 95%CI, 1.01-9.12).

**Conclusions:**

NAFLD development increases the risk of incident diabetes, whereas NAFLD remission reduces the risk of incident diabetes. Moreover, presence of NASH at baseline could attenuate the protective effect of NAFLD remission on incident diabetes. Our study suggests that early intervention of NAFLD and maintenance of non-NAFLD are important for prevention of diabetes.

## Introduction

Type 2 diabetes mellitus (T2DM) poses a serious challenge for human health due to complicated cardiovascular diseases and mortality (1). The prevalence of diabetes is rapidly increased (2, 3), therefore, it is urgent to identify risk factors for incident diabetes in order to prevent major complications. Accumulating evidence has demonstrated that non-alcoholic fatty liver disease (NAFLD) is emerging as a leading cause of chronic liver disease worldwide in the past two decades (4). The close association of NAFLD and diabetes has been well determined. In patients with diabetes the prevalence of NAFLD is as high as 40-70% (5) and NAFLD patients are usually accompanied with impaired glucose metabolism as well (6, 7). A long-term effect of NAFLD on incident T2DM risk has been reported. A 19-year cohort study reported that the risk of T2DM was increased by 11.7 folds in NAFLD subjects as compared to the general population (8). Sinn Dong Hyun reported that NAFLD subjects with either normal weight or overweight/obesity was an independent risk for incident diabetes (9). Of note, the co-existence of NAFLD and diabetes results in worse hepatic injury, as the presence of diabetes accelerates the progression of simple fatty liver to steatohepatitis, cirrhosis, and hepatocellular carcinoma (10). Moreover, unfavorable extrahepatic disease risks should be highlighted. The co-existence of NAFLD in patients with diabetes leads to an increased risk of chronic kidney disease (1.87-fold), cardiovascular disease (1.96-fold), and cardiovascular mortality (3.46-fold), imposing a heavy burden on global healthcare systems (11–14).

NAFLD can be dynamic across the lifespan, changing from remission to worsening. As the pathophysiology of the association between NAFLD development and incident diabetes has been well illustrated, which involves insulin resistance, increased lipogenesis, overproduced hepatic glucose, and dysregulated hepatokines thus contributing to β-cell dysfunction, the change in NAFLD status might modify the risk of diabetes (15, 16). Several previous studies have proved that the risk of incident diabetes was increased with the development of fatty liver and worsening of fatty liver (17). However, the effect of remission of NAFLD on incident diabetes has not been well studied. As NAFLD could be ameliorated by clinical intervention (18, 19), targeting the effect of the change in NAFLD, especially the improvement of NAFLD might be important for diabetes prevention.

In the present study, we explored whether the development and remission of NAFLD increased and reduced the risk of incident diabetes in a prospective cohort.

## Materials and methods

### Subjects and study design

Our cohort study was conducted in the Chongming District, Shanghai and the detailed information about study design, eligibility criteria, and sampling has been described previously (20). In brief, a total of 9930 participants received a baseline survey from 2011 to 2012 and 7707 participants completed the follow-up survey in 2014. In our present study, 3577 subjects who had complete baseline and follow-up information were included. Those individuals with diabetes at baseline (n=771), a history of known liver disease including viral or autoimmune hepatitis, liver cancer, or cirrhosis (n=35), abusing alcohol (alcohol consumption >140 g/week in men or >70 g/week in women, n=75), or missing information of fatty liver (n=6) were excluded. Finally, 2690 participants were included for this analysis. Our prospective cohort study was approved by the Ethical Committee of Zhongshan Hospital, Fudan University, and each participant was provided with a written informed consent.

### Clinical and laboratory evaluation

Standard questionnaires were employed to obtain the information about demographic characteristics, lifestyles, history of diseases and medication on site conducted by trained investigators. Body weight and height were obtained in light clothes and bare feet to the nearest 0.1 kg and 0.1 cm, respectively. Body mass index (BMI) was derived from weight in kilograms divided by square of height in meters. Waist circumference (WC) was measured at the level of umbilicus in a standing position. Blood pressure was measured on non-dominant arm at a seated position, three times consecutively with 1-min rest and 10-min interval using an automated electronic sphygmomanometer (OMRON Model HEM-752 FUZZY’ Omron Co., Dalian, China). The average value of three readings was used. Current smokers were defined as participants regularly consuming cigarettes (duration> 6 months) right before the survey. Former smokers were defined as participants with a history of cigarettes consuming for longer than 6 months and having quitted smoking at the time of survey. Similarly, current drinkers were defined as participants regularly consuming alcohol (duration > 6 months) right before the survey. Former drinkers were defined as participants with a history of alcohol consuming for longer than 6 months and having quitted drinking at the time of survey.

Blood samplings were done two times, one at baseline and another at the 3.5-year follow-up. Fasting venous blood samples were collected after at least 10-h fasting. Serum triglyceride (TG), total cholesterol (TC), low density lipoprotein cholesterol (LDL-C), high density lipoprotein cholesterol (HDL-C), alanine aminotransferase (ALT), alanine aminotransferase (AST), gamma-glutamyl transpeptidase (GGT) were measured on the auto analyser (Modular E170, Roche).

### Diabetes definition

A 75g oral glucose tolerance test (OGTT) was conducted and blood samples at 0h and 2h after glucose load were collected. Fasting blood glucose (FBG) and 2-h post-load glucose levels were measured using glucose oxidase method on an auto analyser (Modular P800, Roche). Serum insulin was measured by an electrochemiluminescence assay (Modular E170, Roche). The homeostasis model assessment of insulin resistance index (HOMA_IR) was calculated as fasting insulin (μIU/ml) × fasting glucose (mmol/L)/22.5. Glycated hemoglobin (HbA1c) was measured by high-performance liquid chromatography. According to American Diabetes Association 2010 criteria, diabetes mellitus was defined as 1) self-reported doctor-diagnosed diabetes or taking antidiabetic medications, and/or 2) FBG levels ≥ 7.0 mmol/L and/or, 3) 2h post-load glucose levels ≥ 11.1 mmol/L, and/or 4) HbA1c concentration ≥ 6.5% (48mmol/mol). In the absence of unequivocal hyperglycemia, diagnosis requires two abnormal test results from the same sample or in two separate test samples.

### NAFLD definition

NAFLD was diagnosed by ultrasonography with exclusion of a history of known liver diseases. Liver ultrasonography was operated by two specialists who were blinded to clinical data using a high-resolution B-mode tomographic ultrasound system (Esaote Biomedica SpA, Italy) equipped with a 3.5-MHz probe. Fatty liver was defined as the presence of at least two of the following three findings: 1) diffusely increased echogenicity of the liver relative to kidney; 2) ultrasound beam attenuation; 3) poor visualization of intrahepatic structures. The definitions for NAFLD development were absence of NAFLD at baseline and presence of NAFLD at the end of follow-up, NAFLD remission presence of NAFLD at baseline and absence of NAFLD at the end of follow-up, consistent non-NAFLD absence of NAFLD at baseline till the end of follow-up and sustained NAFLD presence of NAFLD at baseline till the end of follow-up. Non-invasive NAFLD scores was used to assess the non-alcoholic steatohepatitis (NASH). Gholam’s model was calculated as 2.627 * ln AST + 2.13 for diabetics, with a cut-off for predicting NASH of 8.22 (21, 22).

### Statistical analysis

Normally distributed continuous variables were presented as means with standard deviations (SDs), whereas skewed distributed continuous variables were presented as geometrical median and interquartile range. Continuous variables were compared by student *t* tests and one-way analysis of variance (ANOVA), whereas skewed distributed variables were compared by Mann Whitney U and Kruskal Wallis tests. Categorical variables were expressed as proportions and compared across groups using chi-square tests or fisher exact test. The unadjusted and multivariate adjusted logistic regression analyses were performed to investigate the odds ratios of new development and remission of NAFLD on the risk of incident diabetes. In the NAFLD remission group, logistic regression analysis was further performed to compare the risk of incident diabetes between subjects with or without steatohepatitis at baseline. Statistical analyses were performed on SPSS version 26 (IBM Corp., Armonk, NY). A two-sided p value less than 0.05 was considered as statistical significance.

## Results

### Baseline characteristics of participants with and without incident type 2 diabetes

The present study included 2690 participants free of diabetes at baseline from 2011 to 2012, and followed up in 2014. Diabetes developed in 484 subjects (18.0%). The baseline characteristics of participants by incident diabetes at follow-up were shown in **Table 1**. Participants who developed diabetes were older (p = 0.006), had higher BMI and WC, higher concentrations of TC, TG (all p < 0.0001) and LDL-C (p = 0.01) at baseline. The incidence of diabetes was 21.5% in subjects with presence of NAFLD at baseline and 16.1% in subjects without NAFLD at baseline (21.5% VS 16.1%, p < 0.0001).

**Table 1:** Baseline characteristics of participants with and without incident type 2 diabetes: demographics and laboratory values.

	Non-Diabetes (n=2206)	Incident Diabetes (n=484)	P value
Age, y	55 ± 8	56± 8	0.006
Gender (male/female)	586/1620 (27%)	150/334 (31%)	0.048
Smoking status, n (%)			
Current smoker	178 (8.1%)	33 (6.8%)	0.36
Former smoker	70 (3.2%)	11 (2.3%)	
Never smoker	1958 (88.8%)	440 (90.9%)	
Drinking status, n (%)			
Current drinker	74 (4.4%)	15 (3.7%)	0.69
Former drinker	230 (15.6%)	50 (14.9%)	
Never drinker	1264 (79.9%)	253 (81.4%)	
BMI, kg/m^2^	24.1 ± 3.3	24.7± 3.4	<0.0001
WC, cm	82.2 ± 9.5	84.0 ± 10.1	<0.0001
SBP, mmHg	126 ± 17	131± 17	<0.0001
DBP, mmHg	79 ± 10	81 ± 10	<0.0001
Lipids			
Total cholesterol, mmol/L	4.40 ± 0.99	4.57 ± 1.05	<0.0001
Triglycerides, mmol/L	1.22 (0.89-1.74)	1.36 (0.97-1.92)	<0.0001
LDL-C, mmol/L	2.48 ± 0.74	2.58± 0.76	0.01
HDL-C, mmol/L	1.19 ± 0.30	1.20 ± 0.32	0.75
NAFLD at baseline (%)			
Yes	741 (33.6%)	203 (41.9%)	<0.0001
No	1465 (66.4%)	281 (58.1%)	

Data are presented as mean ± SD, number and percentage, or median (IQR). Continuous variables were compared by student t tests, skewed distributed variables were compared by Mann Whitney U tests, categorical variables were compared by chi-square tests. A two-sided p value < 0.05 was considered as statistical significance. BMI, body mass index; WC, waist circumference; SBP, systolic blood pressure; DBP, diastolic blood pressure; LDL-C, low density lipoprotein cholesterol; HDL-C, high density lipoprotein cholesterol; NAFLD, non-alcoholic fatty liver disease.

### The association of NAFLD alteration with incident diabetes

**Table 2** showed the change of NAFLD during 3.5-year follow-up. Of 1746 non-NAFLD subjects at baseline, 580 (33.2%) participants developed NAFLD and 1166 (66.8%) was consistently free of NAFLD throughout the follow-up. Of 944 NAFLD subjects at baseline, 150 participants (15.9%) had NAFLD remission and 794 (84.1%) participants had sustained NAFLD. We then investigated the association of NAFLD alteration and incident diabetes. 170 of 1166 (14.6%) participants with consistent non-NAFLD developed diabetes, whereas 184 of 794 (23.2%) participants with sustained NAFLD developed diabetes. In contrast, 111 of 580 (19.1%) subjects with NAFLD development developed diabetes, and 19 of 150 (12.7%) subjects with NAFLD remission developed diabetes.

**Table 2:** (A) NAFLD status at baseline and follow-up.

Baseline NAFLD status	Follow-up NAFLD status		P value
	No NAFLD (n=1316)	NAFLD (n=1374)	
No NAFLD (n=1746)	1166/1746 (66.8%)	580/1746 (33.2%)	<0.0001
NAFLD (n=944)	150/944 (15.9%)	794/944 (84.1%)	

Data are presented as number and percentage. P values was compared among groups using chi-square test. P value < 0.05 was defined as statistically significant.

**(B):** Incident diabetes according to baseline and follow-up NAFLD status

NAFLD status at baseline and follow-up	No. of cases/total	Incidence rate	P value
Sustained non-NAFLD	170/1166	14.6%	0.015
New NAFLD	111/580	19.1%	
NAFLD remission	19/150	12.7%	0.004
Sustained NAFLD	184/794	23.2%	

Data are presented as number and proportion. P values were compared across groups sustained non-NAFLD VS new NAFLD; NAFLD remission VS sustained NAFLD using chi-square tests. P value < 0.05 was defined as statistically significant.

### The risk for incident diabetes according to NAFLD alteration by logistic regression analysis

**Table 3** showed the baseline clinical and biochemical characteristics according to NAFLD alterations during 3.5-year follow-up. The subjects with consistent non-NAFLD were younger by age, had lower BMI, WC, blood pressure, plasma glucose, TG, and higher HDL-C, whereas sustained-NAFLD group was older and had higher BMI, WC, blood pressure, plasma glucose, insulin resistance, and more adverse lipid metabolism at baseline (all p < 0.0001). There were no significant differences in smoking or drinking status across four groups.

**Table 3:** Baseline characteristics of the cohort stratified by NAFLD status at baseline and at follow up.

	NAFLD status				
	Sustained non-NAFLD	New NAFLD	Remission of NAFLD	Sustained NAFLD	P for trend
Age, y	55 ± 8	54 ± 8	56 ± 8	56 ± 7	<0.0001
Gender (male/female, male%)	343/823 (29%)	132/448 (23%)	37/113 (25%)	224/570 (28%)	0.023
Smoking status, n (%)					
Current smoker	97 (8.3%)	45 (7.8%)	8 (5.3%)	61 (7.7%)	0.78
Former smoker	40 (3.4%)	17 (2.9%)	4 (2.7%)	20 (2.5%)	
Never smoker	1029 (88.3%)	518 (89.3%)	138 (92.0%)	713 (89.8%)	
Drinking status, n (%)					
Current drinker	50 (4.3%)	24 (4.1%)	4 (2.7%)	38 (4.8%)	0.90
Former drinker	177 (15.2%)	94 (16.2%)	21 (14.0%)	125 (15.7%)	
Never drinker	939 (80.5%)	462 (79.7%)	125 (83.3%)	631 (79.5%)	
BMI, kg/m^2^	22.4 ± 2.5	24.5 ± 2.7	25.1 ± 2.7	26.7 ± 3.2	<0.0001
WC, cm	78 ± 8	83± 8	86 ± 9	89 ± 8	<0.0001
SBP, mmHg	124 ± 18	126 ± 17	127 ± 17	131 ± 17	<0.0001
DBP, mmHg	77 ± 10	79 ± 9	80 ± 10	82 ± 10	<0.0001
Lipids					
Total cholesterol, mmol/L	4.37 ± 0.99	4.34 ± 0.99	4.55 ± 1.12	4.56 ± 1.00	<0.0001
Triglycerides, mmol/L	1.01 (0.78-1.40)	1.25 (0.92-1.82)	1.34 (0.98-1.81)	1.64 (1.20-2.31)	<0.0001
LDL-C, mmol/L	2.46 ± 0.74	2.43 ± 0.69	2.62 ± 0.85	2.58 ± 0.75	<0.0001
HDL-C, mmol/L	1.27 ± 0.32	1.15 ± 0.28	1.15 ± 0.29	1.11 ± 0.26	<0.0001
FBG, mmol/L	5.52 ± 0.51	5.56 ± 0.53	5.73 ± 0.54	5.72 ± 0.53	<0.0001
2h-BG, mmol/L	6.56 ± 1.62	7.03 ± 1.54	7.00 ± 1.68	7.59 ± 1.58	<0.0001
HbA1c, %	5.66 ± 0.37	5.71 ± 0.36	5.75 ± 0.35	5.81 ± 0.35	<0.0001
HbA1c, mmol/mol	38 ± 4.1	39 ± 4.0	39 ± 3.8	40 ± 3.8	<0.0001
HOMA_IR	1.32 (0.96-1.71)	1.63 (1.31-2.19)	1.79 (1.29-2.33)	2.33 (1.74-2.93)	<0.0001

Data are presented as mean ± SD, number and percentage, or median (IQR). Continuous variables were compared by one-way analysis of variance (ANOVA). Skewed distributed variables were compared by Kruskal Wallis tests. Categorical variables were compared by chi-square tests. A two-sided p value < 0.05 was considered as statistical significance. BMI, body mass index; WC, waist circumference; SBP, systolic blood pressure; DBP, diastolic blood pressure; LDL-C, low density lipoprotein cholesterol; HDL-C, high density lipoprotein cholesterol; NAFLD, non-alcoholic fatty liver disease.

**Figure 1:**
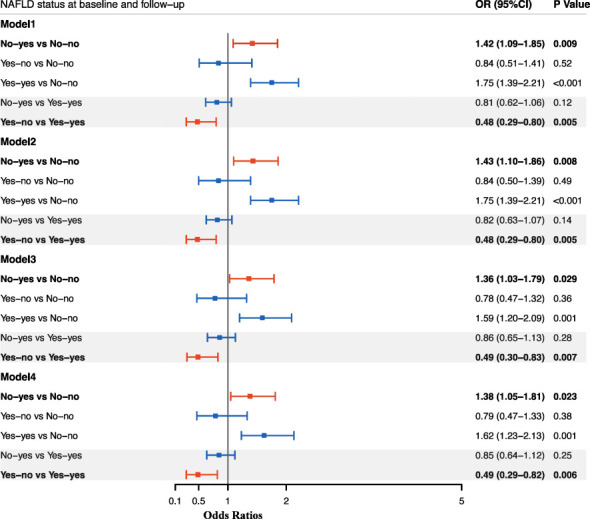
Odds ratios for incident diabetes according to change in NAFLD status between baseline and follow-up. Data are presented as Odds ratios (ORs), and the corresponding 95%CI in each group. Logistic regression models were used to estimate the ORs, 95% CIs, and P values. Model 1: adjusted for age, gender; Model 2: adjusted for age, gender, smoking and drinking status; Model 2: adjusted for age, gender, smoking and drinking status, baseline BMI; Model 4: adjusted for age, gender, smoking and drinking status, baseline WC. No-yes: absence of NAFLD at baseline but presence of NAFLD at follow-up; No-no: absence of NAFLD at baseline till the follow-up; Yes-no: presence of NAFLD at baseline but absence of NAFLD at follow-up; Yes-yes: presence of NAFLD at baseline till the follow-up.

**Figure 2:**
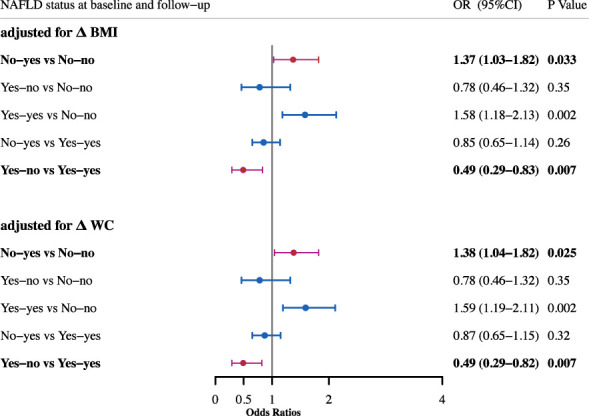
Odds ratios for incident diabetes after adjustment for change of BMI and WC. All adjusted for age, sex, smoking and drinking status, baseline BMI and baseline WC.

Then logistic regression analyses were performed to study the effect of NAFLD alteration on the risk of incident diabetes (**Figure 1**). After adjustment for age, gender, smoking and drinking status, subjects with NAFLD development had a significantly higher risk for diabetes as compared with sustained non-NAFLD group (OR, 1.43; 95%CI, 1.10-1.86). The risk was not changed after further adjustment for BMI (OR, 1.36; 95%CI, 1.03-1.79) or WC (OR, 1.38; 95%CI, 1.05-1.81). After adjustment for age, gender, smoking and drinking status, subjects with remission of NAFLD had a significantly decreased risk for diabetes as compared with sustained NAFLD (OR, 0.48; 95%CI, 0.29-0.80). The decreased risk was not changed after further adjustment for BMI (OR, 0.49; 95%CI, 0.30-0.83) or WC (OR, 0.49; 95%CI, 0.29-0.82). Since change in NAFLD status is always accompanied with change of BMI or WC, and meanwhile BMI and WC have strong associations with incident diabetes, therefore we assessed the risk after adjustment for BMI change and WC change in the existing model, respectively. The results showed that the association of change of NAFLD status with incident diabetes was independent of the change of BMI and WC (**Figure 2**).

### The association between baseline NAFLD severity and risk of incident diabetes in NAFLD remission group

In order to investigate what might contribute to the incidence of diabetes in subjects whose NAFLD remitted, we then calculated the Gholam’s model to assess their NAFLD severity at baseline. 27 of 150 (18.0%) subjects were identified with NASH at baseline. The incidence of diabetes in subjects with NASH at baseline was obviously higher than in those without NASH at baseline (25.9% VS 9.8%, p=0.048) (**Supplemental Table 1**). In the age, gender adjusted- logistic model, presence of NASH at baseline increased risk of incident diabetes in participants with NAFLD remission (OR, 3.08; 95%CI, 1.05-8.99). After further adjustment for smoking and drinking status, and baseline BMI, the association persisted (OR, 3.03; 95%CI, 1.01-9.12) (**Table 4**).

**Table 4:** Odds ratios for incident diabetes according to Gholam’s model assessment at baseline in the NAFLD remission group.

NAFLD severity at baseline	No. of cases/controls	Model 1		Model 2	
		OR (95% CI)	P value	OR (95% CI)	P value
Gholam’s <8.22	7/20	Ref.		Ref.	
Gholam’s >8.22	12/111	3.08 (1.05-8.99)	0.040	3.03 (1.01-9.12)	0.048

Model1: adjusted for age, gender;

Model2: adjusted for age, gender, smoking and drinking status, baseline BMI.

## Discussion

NAFLD is indicative of intrahepatic triglyceride accumulation and strongly associated with diabetes (15) and cardiovascular disease (23). Previous studies have indicated that NAFLD patients were more likely to have impaired glucose regulation and to develop type 2 diabetes (5, 6). Park SK et al. have revealed that, compared to non-NAFLD participants, mild to moderate NAFLD patients increased the risk of incident diabetes by 42% and moderate to severe NAFLD increased the risk of incident diabetes by 158% in 5-year follow-up. The associations were independent of age, BMI, smoking status, regular exercise or family history of diabetes (24). Given that liver fat content is variable, NAFLD status can change from remission to worsening. As the pathophysiology of the interplay between NAFLD and incident diabetes has been elucidated, the change in NAFLD status might modify the risk of incident diabetes. However, the association of the change of NAFLD status, especially the NAFLD remission with incident diabetes has not been well studied.

Our present study showed that new development of NAFLD increased the incident diabetes, in accordance with previous studies (25, 26). Yamazaki H et al. reported that NAFLD remission reduced the risk of incident diabetes (25), whereas, the association was not observed by Sung KC et al., probably due to they adopted different controls, the former focused on whether NAFLD remission reduced the risk of incident diabetes, and the latter focused on whether people had an increased risk of diabetes even if NAFLD resolved (27). In our study, NAFLD remission markedly decreased the incident diabetes compared with sustained NAFLD. Since NAFLD status was changeable, and NAFLD remission reduced risk of incident diabetes, targeting the improvement of NAFLD might be important to prevent diabetes. NAFLD could be ameliorated by lifestyle intervention, including lifestyle modification and physical exercise, medications, and bariatric surgery as well (18, 19, 28, 29). Petersen KF et al. reported that 8% of body weight loss by caloric restriction could reverse NAFLD and hepatic insulin resistance and further normalized plasma glucose levels in patients with diabetes (30). Taylor R et al. demonstrated that removal of excess intrahepatic fat *via* substantial weight loss can normalize hepatic insulin responsiveness, which was required remission in human type 2 diabetes (31). They revealed that both fatty liver and diabetes were closely associated with hepatic insulin resistance and speculated that fatty liver played a central role in the progression of diabetes (32).

Our data indicated that remission of NAFLD reduced the risk of incident diabetes, which might be explained by: 1) the improvement of hepatic insulin resistance; 2) alteration of hepatokine production, such as a reduction of fetuin A levels (33). Liver fat content is an important regulator of hepatic insulin sensitivity, and hepatic insulin sensitivity was found to be a strong predictor of glucose tolerance. And decreased liver fat is always accompanied by a decrease in serum Fetuin A levels. Fetuin A can induce insulin resistance by interruption of insulin receptors and activation of toll-like receptors (34).

However, there were still a proportion of subjects developing diabetes even though their NAFLD remitted. A meta-analysis in 501,022 adult individuals showed that patients with more ‘severe’ NAFLD were also more likely to develop incident diabetes (17). Similarly, we found in participants with NAFLD remission, those predicted to have NASH at baseline were more likely to develop diabetes. This indicated that increased severity of NAFLD (ie. NASH) at baseline could attenuate the protective effect of NAFLD remission. Therefore, early intervention of NAFLD is important.

### Strengths and limitations

The strengths of the present study are as follows. First, we focused on change in NAFLD status as effects of alcohol consuming and other liver diseases were ruled out. We conducted a well-designed longitudinal cohort and reported the effect of NAFLD status change, including new development and remission of NAFLD on incident diabetes in a 3.5-year Chinese cohort population for the first time. Third, standardized collection of covariates allowed for adjustment for potential confounders. We also have some limitations. First, the study was performed in middle-aged and older Chinese population and cannot be generalized to adolescent or other ethnical populations. Second, NAFLD was determined by ultrasonography, which had limited sensitivity to detect low-level liver fat, limiting the generalizability of our study to earlier stages of NAFLD. NASH were assessed by non-invasive score instead of gold-standard hepatic biopsy. Third, diagnoses of diabetes and NAFLD were only made at baseline and the 3.5-year follow-up, so it might not differentiate which one developed first, and an annual screening for incident diabetes could be helpful.

## Conclusion

In conclusion, the change of NAFLD is associated with the change of risk of diabetes. NAFLD development increases the risk of incident diabetes, whereas NAFLD remission decreases the risk of incident diabetes, after adjustment for multiple potential confounders. Moreover, presence of NASH at baseline could attenuate the protective effict of NAFLD remission on incident diabetes. Therefore, our study indicates that early intervention of NAFLD and maintenance of non-NAFLD are important for prevention of diabetes.

## Data availability statement

The original contributions presented in the study are included in the article/**Supplementary Material**. Further inquiries can be directed to the corresponding authors.

## Ethics statement

The studies involving human participants were reviewed and approved by The Ethical Committee of Zhongshan Hospital, Fudan University. The patients/participants provided their written informed consent to participate in this study.

## Author contributions

LQ and XL contributed to the conception and design of the study. ZY, and YF contributed to the acquisition of data. CC, and YZ analyzed the data. CC wrote the manuscript. QS reviewed and revised the manuscript. All authors read and approved the final manuscript. All authors contributed to the article and approved the submitted version.

## Supplementary material

Supplementary Table 1 Changes in clinical parameters of participants stratified by NAFLD status at baseline and at follow up.

Supplementary Table 2 Incident diabetes according to Gholam’s model assessment at baseline in the NAFLD remission group. Data are presented as number and percentage. P values was compared among groups using chi-square test. P value < 0.05 was defined as statistically significant.

Supplementary Table 3 Incident diabetes according to BARD score assessment at baseline in the NAFLD remission group. Data are presented as number and percentage. P values was compared among groups using chi-square test. P value < 0.05 was defined as statistically significant.

Supplementary Table 4 Incident diabetes according to BAAT score assessment at baseline in the NAFLD remission group. Data are presented as number and percentage. P values was compared among groups using chi-square test. P value < 0.05 was defined as statistically significant.
