# Supplementary material for: The change of non-alcoholic fatty liver disease is associated with risk of incident diabetes
Source: Front Endocrinol (Lausanne). 2023 May 4;14:1108442. doi: 10.3389/fendo.2023.1108442 (PMC10194027; doi:10.3389/fendo.2023.1108442)
Supplement: Supplementary Table 1 — Changes in clinical parameters of participants stratified by NAFLD status at baseline and at follow up. [file Table_1.docx]

**Table 1 Changes in clinical parameters of participants stratified by NAFLD status at baseline and at follow up.**

|  | NAFLD status | | | |  |
| --- | --- | --- | --- | --- | --- |
|  | Sustained  non-NAFLD | New  NAFLD | Remission of NAFLD | Sustained NAFLD | P for trend |
| weight change (kg) | 0.10  (-1.70, 1.80) | 1.20  (-0.60,3.20) | -0.90  (-3.50, 0.60) | 0.20  (-1.60, 2.30) | <0.0001 |
| BMI change (kg/m^2^) | 0.04  (-0.68, 0.71) | 0.51  (-0.24,1.25) | -0.35  (-1.33,0.26) | 0.08  (-0.62,0.91) | <0.0001 |
| WC change  (cm) | 1.50  (-2.50, 5.50) | 2.80  (-1.20, 6.80) | 1.00  (-4.70, 5.00) | 1.50  (-2.00, 5.25) | <0.0001 |
| SBP change (mmHg) | 9 ± 26 | 4± 25 | 8 ± 23 | 0 ± 24 | <0.0001 |
| DBP change (mmHg) | 3 ± 15 | 3 ± 14 | 3 ± 15 | -1 ± 15 | 0.016 |

**Table 2 Incident diabetes according to Gholam’s model assessment at baseline in the NAFLD remission group.**

| Baseline NAFLD severity | Follow-up diabetes status | | P value |
| --- | --- | --- | --- |
|  | No diabetes  (n=131) | Diabetes  (n=19) |  |
| Gholam’s <8.22  (n=123) | 111/123  (90.2%) | 12/123  (9.8%) | 0.048 |
| Gholam’s >8.22  (n=27) | 20/27  (74.1%) | 7/27  (25.9%) |  |

Data are presented as number and percentage. P values was compared among groups using chi-square test. P value < 0.05 was defined as statistically significant.

**Table 3 Incident diabetes according to BARD score assessment at baseline in the NAFLD remission group**

| Baseline NAFLD fibrosis | Follow-up diabetes status | | P value |
| --- | --- | --- | --- |
|  | No diabetes  (n=131) | Diabetes  (n=19) |  |
| BARD score 0-2  (n=140) | 122/140  (87.1%) | 18/140  (12.9%) | 0.79 |
| BARD score 3-4  (n=10) | 9/10  (90.0%) | 1/10  (10.0%) |  |

Data are presented as number and percentage. P values was compared among groups using chi-square test. P value < 0.05 was defined as statistically significant.

**Table 4 Incident diabetes according to BAAT score assessment at baseline in the NAFLD remission group**

| Baseline NAFLD fibrosis | Follow-up diabetes status | | P value |
| --- | --- | --- | --- |
|  | No diabetes  (n=131) | Diabetes  (n=19) |  |
| BAAT score 0-2  (n=145) | 126/145  (86.9%) | 19/145  (13.1%) | 0.39 |
| BAAT score 3-4  (n=5) | 5/5  (100.0%) | 0/5  (0.0%) |  |

Data are presented as number and percentage. P values was compared among groups using chi-square test. P value < 0.05 was defined as statistically significant.
